# Hydrolysable Tannins Exhibit Acetylcholinesterase Inhibitory and Anti-Glycation Activities In Vitro and Learning and Memory Function Improvements in Scopolamine-Induced Amnesiac Mice

**DOI:** 10.3390/biomedicines9081066

**Published:** 2021-08-23

**Authors:** Lih-Geeng Chen, Shyr-Yi Lin, Yi-Shan Lee, Ching-Chiung Wang, Wen-Chi Hou

**Affiliations:** 1 Department of Microbiology, Immunology and Biopharmaceuticals, College of Life Sciences, National Chiayi University, Chiayi 600, Taiwan; lgchen@mail.ncyu.edu.tw; 2 Traditional Herbal Medicine Research Center, Taipei Medical University Hospital, Taipei 110, Taiwan; 3 Department of General Medicine, School of Medicine, College of Medicine, Taipei Medical University, Taipei 110, Taiwan; sylin@tmu.edu.tw; 4 Department of Internal Medicine, Division of Gastroenterology, Wan Fang Hospital, Taipei Medical University, Taipei 116, Taiwan; 5 Graduate Institute of Pharmacognosy, Taipei Medical University, Taipei 110, Taiwan; m303103009@tmu.edu.tw; 6 School of Pharmacy, College of Pharmacy, Taipei Medical University, Taipei 110, Taiwan

**Keywords:** acetylcholinesterase, passive avoidance, scopolamine, water caltrop hulls, water maze

## Abstract

Agricultural waste from the hulls of water caltrop (*Trapa taiwanesis* Nakai, TT-hull) was extracted by either steeping them in cold 95% ethanol (C95E), refluxing 95E, refluxing 50E, or refluxing hot water (HW) to obtain C95EE, 95EE, 50EE, and HWE, respectively. These four extracts showed acetylcholinesterase (AChE) inhibitory activities and free radical scavenging activities, as well as anti-non-enzymatic protein glycation in vitro. Eight compounds were isolated from TT-hull-50EE and were used to plot the chromatographic fingerprints of the TT-hull extracts, among which tellimagrandin-I, tellimagrandin-II, and 1,2,3,6-tetra-galloylglucose showed the strongest AChE inhibitory activities, and they also exhibited anti-amyloid β peptide aggregations. The scopolamine-induced amnesiac ICR mice that were fed with TT-hull-50EE or TT-hull-HWE (100 and 200 mg/kg) or tellimagrandin-II (100 and 200 mg/kg) showed improved learning behavior when evaluated using passive avoidance or water maze evaluation, and they showed significant differences (*p* < 0.05) compared to those in the control group. The enriched hydrolysable tannins of the recycled TT-hull may be developed as functional foods for the treatment of degenerative disorders.

## 1. Introduction

The global prevalence of dementia is increasing continuously [1], and it has been ranked the 14th (2000), 8th (2010), and 5th (2016) highest cause of death worldwide [2]. Among different types of dementia, Alzheimer’s disease (AD) was ranked first for around 60–70% of all cases, and the AD populations accounted for 75% and 80%, respectively, of all dementia cases in elderly patients between 80 and 89 years old and older than 90 years [3]. AD is a progressive neurodegenerative disorder, and patients are characterized by dysfunctions in their behaviors, memories, and spatial awareness, and in the late stages, they display an inability to participate in general activities [4]. The neuropathology may involve protein abnormalities of neurofibrillary tangles of tau proteins and senile amyloid plaques of amyloid β peptides (Aβ), neuronal and synaptic losses, and the accompanying lower levels of acetylcholine, inflammation, and oxidative damage [5]. The “amyloid hypothesis” has been the mainstream theory of AD etiology for the past 30 years regarding the imbalance of the production and clearance of Aβ, which aggregates and then accumulates to initiate the progression of AD [5,6]. Researchers have focused on the “amyloid hypothesis” to develop drugs from small molecules such as β-site APP cleaving enzyme 1 (BACE-1) inhibitor and to develop the monoclonal antibodies specific for soluble Aβ, fibrillar Aβ and plaques, or aggregated Aβ; however, none have been successful in clinical trials of AD patients to improve cognitive outcomes [7]. Recently, on 7 June 2021, the FDA approved monoclonal antibody aducanumab, which is specific for aggregated Aβ, as the first treatment to slow down the progression of Alzheimer’s disease; next, post-approval studies will take place for confirmatory trials. The approved therapeutic drugs for clinical AD treatments are only acetylcholinesterase (AChE) inhibitors, which are developed based on the “cholinergic hypothesis”, which suggests that acetylcholine plays a vital role in learning and memory; therefore, dysfunctions in the brain neurons associated with acetylcholine may contribute to cognitive decline. The developed AChE inhibitors showed short-term benefits for improving cognitive symptoms, but they had no effects on the progression of AD [8,9,10,11]. AChE (EC 3.1.1.7) is a cholinergic enzyme that hydrolyzes acetylcholine to terminate neuronal transmission at the postsynaptic neuromuscular junctions. The cholinergic neuron deficits show the classic symptoms of cognitive decline, which are generally found in normal aging and, to a great extent, in AD patients [12,13]. Researchers provide evidence that the acetylcholine levels affected by drugs were correlated with memory function in the elderly and in the efficiency of task learning [9,14]. Scopolamine has been shown to induce amnesia by competitively binding with acetylcholine at muscarinic receptors, and this phenomenon could be recovered by AChE inhibitors. Therefore, the amnesia model induced by scopolamine is generally used to investigate AChE inhibitors and their effects on AD management [9,14,15].

Reactive oxygen species (ROS) and lipid peroxidation-derived free radicals are associated with aging, cardiovascular diseases, and neurodegenerative diseases [16,17,18]. The ROS may participate in mitochondrial dysfunction and/or mitochondrial autophagy, Aβ aggregation, lipid peroxidation, and microglia activation associated with inflammation [19]. Although epidemiological studies showed the relationship between diets rich in fruits and vegetables and decreased risk of cardiovascular diseases [20], several results implied that dietary polyphenol with antioxidant activity in vitro was not correlated with a direct beneficial effect in vivo; further animal experiments and/or clinical trials will be needed to evaluate this in more detail [16,17,18]. Extracts from the leaves of Eucalyptus globulus showed antioxidant and neuroprotective activities against hydrogen peroxide-induced cell death in SH-SY5Y cell models [21]. The electron chain reactions for energy production in mitochondria and non-enzymatic protein glycation may be the two main ROS generation routes in cells. The non-enzymatic protein glycation could irreversibly generate advanced glycation end-products (AGEs), such as well-characterized N^ε^-(carboxymethyl)lysine (CML) [22,23] via Schiff’s base, and Amadori adducts and rearrangements. AGEs have been shown to promote ROS production or enhance TNF-α and IL-6 production, respectively, via the receptor for AGE (RAGE) or NF-κB signaling pathways [24]. It was reported that AGEs were detected in AD amyloid plaques and that the oxidation of glycated proteins might accelerate Aβ accumulation and consequently increase oxidative stress [25].

The water caltrop (*Trapa* *taiwanesis* Nakai, TT), also called water chestnut, is coated by a tough hull and the inside is an edible fruit. In Taiwan, fresh TT fruits are used as vegetables or foods, and steamed TT fruits are eaten as between-meal snacks. In the ancient pharmacopoeia of Chinese Traditional Medicine (Ben Cao Gang Mu and Ben Cao Shi Yi), it was documented that TT fruits are a source of medicine and food for nourishing the spleen and stomach, strengthening the knees, and invigorating and replenishing Qi. The TT-hulls can be applied for external uses; for example, hot-water-extracted TT hulls can be used to dye beards and hair, and the powders of burned TT hulls mixed with oil can be used to treat impetigo, unknown swelling, and herpetic whitlow fingers. The TT-hull decoction in the modern pharmacopoeia of Chinese Materia Medica is described either for internal use to relieve diarrhea and manage dysentery and gastric ulcers, or for external uses to wash injured areas of the body in order to promote healing. In the Guantian District in Taiwan, the main TT production area is near Tainan city, where the TT fruits are harvested in the late autumn, and the vast amounts of TT-hull have become an environmental issue as they are burned or discarded in the local downtown area. Recently, the agricultural waste of the TT-hulls has been successfully recycled to produce valuable new materials, such as macroporous carbon biochars [26] and activated biochars [27], for a wide range of applications. The micronized TT-hulls are reported to possess increased hydration properties and oil-holding capacities, which will provide new functional properties; they can be used to process dietary fibers in food applications [28]. It is reported that the polyphenol fractions and three isolated compounds (eugeniin, 1,2,3,6-tetra-O-galloyl-β-D-glucopyranose, and trapain) from 60% acetone extracts of Japanese water caltrop hulls exhibited antioxidant, in vitro inhibitory activities against α-amylase and α-glucosidase, as well as blood glucose-lowering activities, in an oral carbohydrate tolerance test of fasting mice [29]. In this study, the enriched hydrolysable tannins of the recycled TT-hulls were investigated, as well as their potential use as functional foods for degenerative disorders, through in vitro and in vivo animal experiments.

## 2. Experimental Section

### 2.1. Chemical and Reagents

The acetylthiocholine iodide, dimethylsulfoxide (DMSO), 5′,5′-dithiobis(2-nitrobenzoic acid) [DTNB], galactose, 1,1,1,3,3,3-hexafluoroisopropanol (HFIP), horse radish peroxidase-conjugated goat anti-rabbit IgG (A6154), phosphate-buffered saline (PBS), scopolamine hydrobromide, and thioflavin T (ThT) were purchased from Sigma Chemical Co. (St. Louis, MO, USA). The OxiSelect™ Assay Kit (STA-345) was from Cell Biolabs Inc. (San Diego, CA, USA). The glutathione (GSH) was measured using glutathione assay kits (No. 703002; Cayman Chemical Co., Ann Arbor, MI, USA). The BCA ^TM^ protein assay kit and bovine serum albumin (BSA) were from Thermo Fisher Scientific Inc. (Rockford, IL, USA). The Aβ (1–42) peptide was from Kelowna International Scientific Inc. (New Taipei, Taiwan) with purity higher than 95%. The recombinant human AChE (7574-CE, 10 μg, specific activity of 500 nmol/min/μg) was purchased from R&D Systems Inc. (Minneapolis, MN, USA). The donepezil hydrochloride (D4099) was from Tokyo Chemical Industry Co. (Tokyo, Japan). The anti-CML antibody (ab27684) was from Abcam Inc. (Cambridge, MA, USA).

### 2.2. Crude Extract Preparation

The dried TT-hulls were provided by a farmer from Guantian District, Tainan city, Taiwan. The dried, powdered TT-hulls were extracted by either steeping them in cold 95% ethanol (C95E), refluxing 95E, refluxing 50E, or refluxing hot water (HW) to obtain C95EE, 95EE, 50EE, and HWE, respectively. The 3-L C95E was added to one kg powdered TT-hulls and placed at room temperature for one week. After being filtered, the residue was extracted following the same procedure twice. The filtrates were collected and concentrated as C95EE. Each of 3.5-L of 95E, 3.5-L of 50E, or 5-L of HW was added to 700 g powdered TT-hulls and extracted under refluxing for 3 h. Each filtrate was collected, concentrated, and lyophilized as 95EE, 50EE, and HWE, respectively. The recovery of C95EE, 95EE, 50EE, and HWE, respectively, was approximately 8.6%, 6.5%, 10.2%, and 12.8%.

### 2.3. Isolation of Purified cCmpounds

The 50EE (77 g) was dissolved in water and chromatographed over a Diaion HP-20 column (10.5 cm i.d. × 35 cm) and then eluted with 5-L of H_2_O and MeOH sequentially, which were collected, concentrated, and lyophilized for further purification. The MeOH eluates (26 g) were chromatographed on a Toyopearl HW-40 (C) column (2.5 cm i.d. × 55 cm) and eluted batchwise with one liter of H_2_O, 50% MeOH, 60% MeOH, 70% MeOH, 80% MeOH, and 70% acetone to obtain T-H, T-50M, T-60M, T-70M, T-80M, and T-70A fractions, respectively. The T-50M fraction was purified using a LiChroprep RP-18 column (2.5 cm i.d. × 50 cm) and eluted with 0.05% trifluoroacetic acid (TFA):acetonitrile (CH_3_CN) (92:8) to obtain 1,6-di-O-galloyl-β-D-glucopyranoside (TT-5, 100 mg) and gallic acid (TT-6, 64 mg). The T-60M fraction was purified with a LiChroprep RP-18 column (2.5 cm i.d. × 50 cm) and eluted with 0.05% TFA:CH_3_CN (90:10) to obtain 1,2,3-tri-O-galloyl-β-D-glucopyranoside (TT-3, 105 mg) and 1,2,6-tri-O-galloyl-β-D-glucopyranoside (TT-4, 322 mg). The T-70M fraction was purified with a LiChroprep RP-18 column (2.5 cm i.d. × 50 cm) and eluted with 0.05% TFA:CH_3_CN (82:18) to obtain 1,2,3,6-tetra-O-galloyl-β-D-glucopyranoside (TT-2, 549 mg). The T-80M fraction was purified with a LiChroprep RP-18 column (2.5 cm i.d. × 50 cm) and eluted with 0.05% TFA:CH_3_CN (83:17) to obtain 1,2,3,4,6-penta-O-galloyl-β-D-glucopyranoside (TT-7, 77 mg) and tellimagrandin I (TT-8, 131 mg). The T-70A fraction was purified with a LiChroprep RP-18 column (2.5 cm i.d. × 50 cm) and eluted with 0.05% TFA:CH_3_CN (85:15) to obtain tellimagrandin II (TT-1, 117 mg). The purity of each compound was determined by HPLC and was shown to exceed 95%. All structures were determined by ^1^H-NMR and ^13^C-NMR, including 2D-NMR techniques, and by comparison of those data with authentic compounds as follows.

(a) Tellimagrandin II (TT-1):

^1^H-NMR (500MHz, acetone-d_6_), δ, 7.12 (2H, s, galloyl H), 7.01 (2H, s, galloyl H), 6.97 (2H, s, galloyl H), 6.66 (1H, s,), 6.48 (1H, s,), 6.20 (1H, d, J = 8.4 Hz, Glc H-1), 5.84 (1H, t, J = 9.6Hz, Glc H-3), 5.60 (1H, dd, J = 8.4, 9.6 Hz, Glc H-2), 5.36 (1H, dd, J = 6.6, 13.4 Hz, Glc H-6), 5.22 (1H, t, J = 9.6 Hz, H-4), 4.55 (1H, dd, J = 6.2, 9.6 Hz, Glc H-5), 3.89 (1H, d, 13.4 Hz, Glc H-6). ^13^C-NMR (125 MHz, acetone-d_6_), δ, 167.3, 166.9, 165.5, 164.9, 164.2 (C=O), 145.3, 145.1, 144.9, 144.4, 144.3, 143.6, 138.9, 138.5, 138.3, 135.7, 135.5, 125.6, 125.0, 119.6, 119.5, 118.9, 114.9, 114.8, 109.4, 0.9.3, 109.2, 107.3, 107.0, 92.8 (Glc C-1), 72.4 (Glc C-3), 72.2 (Glc C-5), 71.0 (Glc C-2), 69.9 (Glc C-4), 62.2 (Glc C-6).

(b) 1,2,3,6-tetra-O-galloyl-β-D-glucopyranoside (TT-2):

^1^H-NMR (500MHz, acetone-d_6_), δ, 7.16 (2H, s, galloyl H), 7.080 (2H, s, galloyl H), 7.076 (2H, s, galloyl H), 7.00 (2H, s, galloyl H), 6.15 (1H, d, 8.2 Hz, Glc H-1), 5.67 (1H, t, 9.6 Hz, Glc H-3), 5.47 (1H, dd, J = 8.2, 9.6 Hz, Glc H-2), 4.63 (1H, dd, J = 2.0, 12.0 Hz, Glc H-6), 4.52 (1H, dd, J = 4.8, 12.0 Hz, Glc H-6), 4.15 (1H, ddd, J = 2.0, 4.8, 9.6 Hz, Glc H-5), 4.08 (1H, t, J = 9.6 Hz, Glc H-4). ^13^C-NMR (125 MHz, acetone-d_6_), δ, 166.9, 166.5, 166.2, 165.3(C=O), 146.2, 146.1, 146.0 (galloyl C-3, 5), 139.8, 139.3, 139.0 (galloyl C-4), 121.6, 121.3, 120.6, 120.0 (galloyl C-1), 110.3, 110.19, 110.16, 110.0 (galloyl C-2, 6), 93.6 (Glc C-1), 76.2 (Glc C-5), 76.0 (Glc C-3), 72.0 (Glc C-2), 69.4 (Glc C-4), 63.9(Glc C-6).

(c) 1,2,3-tri-O-galloyl-β-D-glucopyranoside (TT-3):

^1^H-NMR (500MHz, acetone-d_6_), δ, 7.08 (2H, s, galloyl H), 7.07 (2H, s, galloyl H), 6.99 (2H, s, galloyl H), 6.07 (1H, d, J = 8.4 Hz, Glc H-1), 5.60 (1H, t, J = 9.6 Hz, Glc H-3), 5.39 (1H, dd, J = 8.4, 9.6 Hz, Glc H-2), 3.98 (1H, t, J = 9.6 Hz, Glc H-4), 3.92 (1H, dd, J = 2.0, 12.1 Hz, Glc H-6), 3.82 (1H, dd, 4.8, 12.0 Hz, Glc H-6), 3.78 (1H, ddd, J = 2.0, 4.8, 9.6 Hz, Glc H-5). ^13^C-NMR (125 MHz, acetone-d_6_), δ, 166.6, 166.2, 165.3 (C=O), 146.2, 146.0 (galloyl C-4), 139.7, 139.2, 139.0 (galloyl C-3, 5), 121.4, 120.7, 120.3 (galloyl C-1), 110.3, 110.2, 110.1 (galloyl C-2, 6), 93.5 (Glc C-1), 78.7, 76.3, 72.0, 69.3, 61.9 (Glc C-6).

(d) 1,2,6-tri-O-galloyl-β-D-glucopyranoside (TT-4):

^1^H-NMR (500MHz, acetone-d_6_), δ, 7.14 (2H, s, galloyl H), 7.09 (2H, s, galloyl H), 7.06 (1H, s, galloyl H), 5.95 (1H, d, J = 8.4 Hz, Glc H-1), 5.24 (1H, dd, J = 8.4, 9.4 Hz, Glc H-2), 4.59 (1H, dd, J = 2.0, 12.2 Hz, Glc H-6), 4.44 (1H, dd, J = 4.8, 12.2 Hz, Glc H-6), 3.97 (1H, t, J = 9.4 Hz, Glc H-3), 3.92 (1H, ddd, J = 2.0, 4.8, 9.4 Hz, Glc H-5), 3.73 (1H, t, J = 9.4 Hz, Glc H-4). ^13^C-NMR (125 MHz, acetone-d_6_), δ, 166.9, 166.4, 165.4 (C=O), 146.13, 146.10, 146.0 (galloyl C-4), 139.6, 139.0, 138.9 (galloyl C-3, 5), 121.7, 121.4, 120.3 (galloyl C-1), 110.3, 110.2, 110.0 (galloyl C-2, 6), 93.7 (Glc C-1), 76.2 (Glc C-5), 75.5 (Glc C-3), 74.0 (Glc C-2), 71.2 (Glc C-4), 64.1 (Glc C-6).

(e) 1,6-di-O-galloyl-β-D-glucopyranoside (TT-5):

^1^H-NMR (500MHz, acetone-d_6_), δ, 7.15 (2H, s, galloyl H), 7.11 (2H, s, galloyl H), 5.70 (1H, d, J = 7.4 Hz, Glc H-1), 4.55 (1H, d, J = 2.0, 12.1 Hz, Glc H-6), 4.37 (1H, dd, J = 5.4, 12.1 Hz, Glc H-6), 3.77 (1H, ddd, J = 2.0, 5.4, 8.6 Hz, Glc H-5), 3.62-3.53 (3H, m, Glc H-2, H-3, H-4). ^13^C-NMR (125 MHz, acetone-d_6_), δ, 166.9, 165.8 (C=O), 146.11, 146.07 (galloyl C-4), 139.4, 138.9 (galloyl C-3, 5), 121.7, 121.0 (galloyl C-1), 110.4, 110.0 (galloyl C-2, 6), 95.8 (Glc C-1), 77.8, 76.1, 73.9, 70.9, 64.4 (Glc C-6).

(f) gallic acid (TT-6):

^1^H-NMR (acetone-d_6_, 500MHz), δ, 7.13 (2H, s, galloyl H). ^13^C-NMR (125 MHz, acetone-d_6_), δ, 168.0(C=O), 146.0(galloyl C-4), 138.6(galloyl C-3, 5), 122.1(galloyl C-1), 110.1 (galloyl C-2, 6).

(g) 1,2,3,4,6-penta-O-galloyl-β-D-glucopyranoside (TT-7):

^1^H-NMR (500MHz, acetone-d_6_), δ, 7.17, 7.11, 7.05, 7.01, 6.97 (each 2H, galloyl-H), 6.33 (1H, d, J = 8.3 Hz, Glc H-1), 6.01 (1H, t, J = 9.7 Hz, Glc H-3), 5.65 (1H, t, J = 9.7 Hz, Glc H-4), 5.62 (1H, dd, J= 8.3, 9.7 Hz, Glc H-2), 4.56 (1H, m, Glc H-5), 4.53 (1H, brd, J = 12.0 Hz, Glc H-6), 4.40 (1H, dd, J = 5.0, 12.0 Hz, Glc H-6). ^13^C-NMR (125 MHz, acetone -d_6_), δ, 166.5, 166.0, 165.8, 165.7, 165.1 (C=O), 146.3, 146.1, 146.1, 146.1, 146.0 (galloyl C-3, 5,), 139.9, 139.4, 139.4, 139.2, 139.1 (galloyl C-4), 121.5, 120.8, 120.7, 120.0 (galloyl C-1), 110.5, 110.4, 110.3, 110.2 (galloyl C-2, 6), 93.4 (Glc C-1), 74.0 (Glc C-3), 73.4 (Glc C-5), 71.8 (Glc C-2), 69.4 (Glc C-4), 62.9 (Glc C-6).

(h) Tellimagrandin I (TT-8):

Ratio of α-anomer/β-anomer = 6: 4. ^1^H-NMR (500 MHz, methanol-d_4_), α-anomer, δ, 7.01 (2H, s, galloyl), 6.93 (2H, s, galloyl), 6.61(1H, HHDP), 6.50 (1H, HHDP), 5.83 (1H, t, J = 9.9 Hz, Glc H-3), 5.49 (1H, d, J = 3.8 Hz, Glc H-1), 5.31 (1H, dd, J = 6.8, 12.9 Hz, Glc H-6), 5.11 (1H, m, Glc H-2), 5.09 (1H, m, Glc H-4), 4.64 (1H, ddd, J = 0.9, 6.6, 10.0 Hz, Glc H-5), 3.83 (1H, dd, J = 1.3, 12.9 Hz, Glc H-6). β-anomer, δ, 7.00 (2H, s, galloyl), 6.89 (2H, s, galloyl). 6.61 (1H, HHDP), 6.46 (1H, HHDP), 5.59 (1H, t, J = 9.6 Hz, Glc H-3), 5.34 (1H, dd, J = 6.6, 13.5 Hz, Glc H-6), 5.20 (1H, dd, J = 7.9, 9.6 Hz, Glc H-2), 5.14 (1H, m, Glc H-4), 4.96 (1H, d, J = 7.9 Hz, Glc, H-1), 4.20 (1H, dd, J = 5.9, 10.0 Hz, Glc H-5), 3.91 (1H, d, J = 12.9 Hz, Glc H-6). ^13^C-NMR (125 MHz, methanol-d_4_) δ: 169.9, 169.8, 169.5, 169.4, 168.1, 167.9, 167.6, 167.3, 146.6, 146.5, 146.4, 146.3, 146.1, 146.0, 145.0, 140.3, 140.2, 140.1, 137.8, 126.50, 126.47, 126.1, 126.0, 121.1, 120.9, 120.8, 116.9, 116.8, 116.61, 116.56, 110.56, 110.51, 110.5 (Galloyl C-2, C-6), 108.80(HHDP), 108.76(HHDP), 108.4 (HHDP), 97.3 (β-Glc C-1), 91.9 (α-Glc C-1), 75.0 (β-Glc C-2), 74.5 (β-Glc C-3), 73.8 (α-Glc C-4), 72.9 (β-Glc C-5), 72.1 (β-Glc C-4, α-Glc C-2), 71.8 (α-Glc C-3), 67.8 (α-Glc C-5), 64.43 (β-Glc C-6), 64.36 (α-Glc C-6).

For fingerprinting analysis, each extract was prepared at 10 mg/mL. Analysis was performed using the LiChrospher 100 RP-18e column (4 mm i.d. × 250 mm, 5µm) using a Waters Chromatography system. A gradient elution program of mobile phase was set as follows: 0.05% TFA:CH_3_CN (0 min, 95:5; 45 min, 80:20; 55 min, 80:20; 56 min, 95:5; 66 min, 95:5). The mobile phase was pumped at the flow rate of 1.0 mL/min, and 10 μL was injected for analysis. The column temperature was kept at 40 °C, and the wavelength was set at 280 nm for monitoring.

### 2.4. AChE Inhibitory Activities In Vitro

The acetylthiocholine iodide and DTNB were used for in vitro AChE inhibitory activity assays following the previous report [30]. The total 100 μL solution contained 50 μL of 100 mM phosphate buffer (pH 7.5), the diluted recombinant human AChE (0.079 μg/mL in 100 mM phosphate buffer, pH 7.5), and each extract (62.5, 125, 250, 500, and 1000 μg/mL) or purified compound (TT-1, 6.25–50 μM; TT-2, 25–200 μM; TT-3 and TT-6, 50–400 μM; TT-4, 100–800 μM; TT-8, 12.5–100 μM) in DMSO. An equal volume of DMSO instead of the sample solution was used as a blank and expressed as 100% AChE activity. The absorbance changes were recorded at 405 nm for 10 min. The AChE inhibition (%) was calculated as follows: [(A405_blank_−A405_sample_)/(A405_blank_)] × 100%. The concentration for 50% inhibition (IC_50_) of AChE activity of each TT-hull extract or purified compound was calculated from each linear equation by three test concentrations and their corresponding AChE inhibitions. For C95EE, 95EE, and 50EE, 125, 250, and 500 μg/mL were used; for HWE, 200, 250, and 500 μg/mL were used; for TT–1 and TT–8, 12.5, 25, and 50 μM were used; for TT–2, 50, 100, and 200 μM were used; for TT–3 and TT–6, 100, 200, and 400 μM were used.

### 2.5. Effects of Extracts and Purified Compounds of TT-Hull on Oxygen Radical Absorbance Capacity

The antioxidant index of free radical scavenging activities of the extracts (1.5 or 2.5 μg/mL) and purified compounds (10 μM) of TT-hull were determined by a commercial OxiSelect™ Assay Kit for oxygen radical absorbance capacity (ORAC) determination [31]. The standard curve of ORAC was plotted using different concentrations of water-soluble vitamin E analog (the compound’s name was Trolox, 6-hydroxy-2,5,7,8-tetramethylchroman-2-carboxylic acid) at 2.5, 5, 10, 20, 40, and 60 μM. Therefore, the ORAC activities of TT-hull extracts or purified compounds were calculated equivalently to the Trolox effects and expressed as Trolox equivalents (μM).

### 2.6. Effects of TT-Hull Extracts on Anti-Glycation in Non-Enzymatic BSA/Galactose Models

The non-enzymatic glycation model of BSA/galactose was used following the previous experiments [31,32]. The total 100 μL reaction solution included 2 mg/mL, 20 μL of BSA solution, 1M of 60 μL galactose solution, 10 μL of 10-fold diluted PBS solution, and TT-hull extracts (12.5, 25, 50, 100 μg/mL), which was placed in a 37 °C water bath for 11 days. The BSA only was the blank, and the mixture without sample addition was the control. Each of these mixtures was subjected to 10% sodium dodecyl sulfate–polyacrylamide gel electrophoresis, one was stained for proteins by a Coomassie brilliant blue R-250 solution, and one was then transferred onto polyvinylidene difluoride membranes for immune staining. The anti-CML antibody was used at a 1000-fold dilution and the HRP-conjugated secondary antibody was used at a 5000-fold dilution, and they were detected by the chemiluminescent system (no. WBKL S0050; Immobilon™, Millipore, Burlington, MA, USA) and then imaged by a GeneGnome 5 system (Syngene, Cambridge, UK).

### 2.7. Effects of Purified Compounds of TT-Hull on Inhibition of Aβ (1–42) Aggregation In Vitro

The ThT fluorescent assay was used to monitor the isolated compounds from the TT-hull on inhibition of Aβ (1-42) peptide aggregations [30]. For the preparation of the Aβ stock film, the synthetic Aβ (1-42) peptide was weighed in an Eppendorf tube and dissolved in HFIP to prepare a 1 mM stock solution; then, HFIP solvent was evaporated to form the Aβ (1-42) film. The stock film was dissolved in 100 μL of 60 mM NaOH, and further diluted with 50 mM phosphate buffer (pH7.4, containing 150 mM sodium chloride, 1 mM EDTA, and 0.02% sodium azide). The 125 μL working solution contained 10 μM Aβ (1-42) peptide, purified compounds (10, 20, and 40 μM in DMSO), and 10 μM ThT solution, which was gently shaken at 37 °C for 24 h. The mixture without the sample additions was the control. The fluorescence ratios of Ex_440nm_/Em_486nm_ were recorded at a time of 0 h and 24 h, and changes in fluorescence over a 24 h period were expressed as ΔE. The inhibition of Aβ aggregation (%) was calculated as [(ΔE_control_−ΔE_sample_)/(ΔE_control_)] × 100%.

### 2.8. The Oral Administration of TT-Hull Extracts or Purified Compounds in Scopolamine-Induced Amnesiac ICR Mice

The oral administration of TT-hull extracts or purified compounds in scopolamine-induced amnesiac ICR mice was performed according to the previous report [30], with some modifications. The 8-week-old male ICR mice were purchased from the Laboratory Animal Center, College of Medicine, National Taiwan University (Taipei, Taiwan). Normal diets (Prolab^®^ RMH2500, Jefferson City, MO, USA) and water were provided during the animal experiments. All animal experimental protocols were approved by the Institutional Animal Care and Use Committee of Taipei Medical University (LAC-2016-0275). After one week of acclimation, for treatments of TT-hull extracts to test prevention, the mice were randomly divided into seven groups (6 heads/group), including the blank, the control, the donepezil-positive control, and the four extract-administered groups. The ICR mice were pre-administered orally with 50EE or HWE at concentrations of 100 and 200 mg/kg once a day by gavage from day 1 to day 10; for the blank, the control, and the donepezil (5 mg/kg)-positive control, the mice received an equal volume of distilled water for parallel experiments. From day 11 to day 20, the same procedure was performed in the 50EE- and HWE-administered groups once a day by gavage; in the blank and the control groups, the same equal volume of distilled water was orally administered once a day by gavage; in the donepezil-positive control, the mice were administered orally once a day with donepezil (5 mg/kg). Except for the blank group, the mice in each group were intraperitoneally injected with scopolamine (1 mg/kg) 30 min after the oral sample administration, and the mice in the blank group were injected with an equal volume of PBS. After being injected with scopolamine or PBS for another 30 min, each mouse was evaluated by a passive avoidance test at day 13 to day 14 and by the Morris water maze from day 17 to day 19. At the end of the experiments at day 20, the mice were sacrificed. For oral administration of purified compounds to test prevention, the ICR mice were pre-administered orally with TT-1 (100 mg/kg, 200 mg/kg) or TT-2 (300 mg/kg) once a day by gavage from day 1 to day 5; for the blank, the control, and the donepezil (5 mg/kg)-positive control, the mice received an equal volume of distilled water for the parallel experiments. From day 6 to day 11, the procedure was the same for the oral administration of the TT-hull extracts. Each mouse was evaluated by a passive avoidance test at day 6 to day 7 and by the Morris water maze from day 8 to day 10. At the end of the experiments at day 11, the mice were sacrificed, and the brain tissue was isolated and stored at −80 °C for further measurements.

### 2.9. Analyses of Learning and Memory Functions in Scopolamine-Induced Amnesiac ICR Mice

The passive avoidance test and Morris water maze were used to evaluate the learning and memory functions of scopolamine-treated mice after the treatments with TT-hull extracts or purified compounds, by following the previous report [30]. The apparatus of the passive avoidance test included a light box and a dark box separated by a guillotine door in a shuttle chamber (AccuScan Instruments Inc, Columbus, OH, USA), and a wired metal floor was placed in the dark box to provide electric foot shocks during the acquisition trial. On the first day of the passive avoidance test (as the acquisition trial), each mouse was acclimatized to the shuttle chamber for 30 min, and then placed in the light box. The mouse entered the dark box, the guillotine door was immediately closed, and, at the same time, an electric shock (0.3 mA) was delivered for 3 sec to the mouse via the wired metal floor. The time for which each mouse stayed in the light box (step-through latency of the acquisition trial, sec) was recorded. If the mouse did not enter the light box until 300 s, the mouse was forced to enter the dark box and was treated with the same electric foot shocks and then sent back to the cage. On the second day of the passive avoidance test (as the retention trial), the same protocol was performed, except that there were no electric foot shocks in the dark box, and the time for which each mouse stayed in the light box (step-through latency of the retention trial) was recorded. For the Morris water maze evaluations, a three-successive-day training protocol was performed; each mouse was trained for 1 min to find and climb onto the platform twice a day at 30 min intervals. For the second training procedure of the third day (as the reference memory task), the time required for the mouse to find and climb onto the platform from the starting point was recorded as the latency (sec). The platform was then removed (as the probe test), and the swimming time and crossing time in the platform quadrant were recorded for each mouse.

### 2.10. AChE Activities and Reduced GSH Levels in Brain Tissue Extracts

At the end of the TT-1 and TT-2 treatments, mice were sacrificed, and the whole brain of each ICR mouse was isolated and immediately stored at −80 °C for the measurement of residual AChE activity and the reduced GSH levels. The whole brain of each mouse was ground into powder by means of a mortar and pestle in liquid nitrogen and then extracted by adding 1 mL of 100 mM phosphate buffer (pH 7.5) to the ice bath. The residual AChE activity determination followed the previous report [30], except that the donepezil (final concentration 100 nM) was premixed with each diluted brain extract for 30 min as the sample, in parallel with the sample determination. The residual AChE activity of each brain extract was expressed as [A405_sample_−A405_sample blank_]/μg protein. The glutathione assay kit (no. 703002; Cayman Chemical Co.) was used to determine the reduced form GSH levels based on the reaction of GSH with DTNB reagents to generate a yellowish 5-thio-2-nitrobenzoic acid and expressed as A412 nm/μg protein.

### 2.11. Statistical Analyses

The quantitative data were presented as mean ± SD of three independent experiments. Multiple group comparisons were performed using one-way analysis of variance (ANOVA) and the *post hoc* Tukey’s test. The different lowercase letters in the anti-Aβ peptide aggregates of purified compounds under the fixed ratio (10:10, 10:20, 10:40) treatment, the different uppercase letters in step-through latency of the acquisition trial, or the different lowercase letters in the retention trial were considered significantly different among groups (*p* < 0.05). The two-group comparison was analyzed using Student’s t-test, and any difference in comparison with the control group was considered statistically significant when *p* < 0.05 (*), or *p* < 0.01 (**), or *p* < 0.001 (***). Statistical analysis was performed using the GraphPad Prism 5.0 software (San Diego, CA, USA).

## 3. Results

### 3.1. Effects of TT-Hull Extracts on AChE Inhibitory Activities, ORAC, and Anti-Non-Enzymatic Glycation Activities

**Figure 1:**
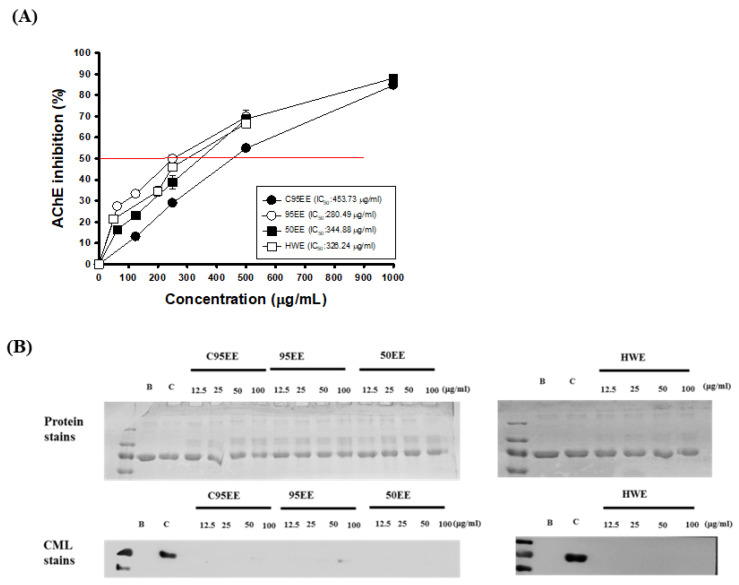
Effects of different TT-hull extracts on (**A**) acetylcholinesterase (AChE) inhibitory activities; (**B**) anti-non-enzymatic glycation in bovine/galactose system by protein staining and immune staining. The TT-hulls were extracted by steeping them in cold 95% ethanol (C95E), refluxing 95E, refluxing 50E, or refluxing hot water (HW) to obtain C95EE, 95EE, 50EE, and HWE, respectively. The quantitative data were presented as mean ± SD of three independent experiments.

Figure 1A shows the effects of four TT-hull extracts, C95EE, 95EE, 50EE, and HWE, on the AChE inhibitory activities in vitro. It was found that the four TT-hull extracts showed dose-dependent AChE inhibitory activities. The IC_50_ of AChE activity of C95EE, 95EE, 50EE, and HWE, respectively, were calculated to be 453.7, 280.5, 344.9, and 326.2 μg/mL in the present assays. The order of AChE inhibition was 95EE > HWE > 50EE > C95EE. The ORAC values of the four TT-hull extracts of C95EE (1.5 μg/mL), 95EE (1.5 μg/mL), 50EE (1.5 μg/mL), and HWE (2.5 μg/mL) in vitro, respectively, were 3.3, 2.8, 3.0, and 3.3 μM Trolox equivalents. Therefore, the order of ORAC antioxidant activities of TT-hull extracts was C95EE > 50EE > 95EE > HWE. Figure 1B shows the effects of TT-hull extracts (12.5, 25, 50, and 100 μg/mL) on CML formations in the BSA/galactose models. There was no significant difference among protein stains of the blank, control, and different amounts of sample addition. However, a clear-stained CML band was found in the control, and little was found in the same position of the blank and sample additions. Based on the photograms of protein stains and immune stains of the CML formations, it was clear that the TT-hull extracts showed anti-glycation activities in the non-enzymatic BSA/galactose models.

### 3.2. Effects of the Isolated Compounds from 50EE on AChE Inhibitory Activities, ORAC, and Anti-Aβ (1-42) Aggregations

**Figure 2:**
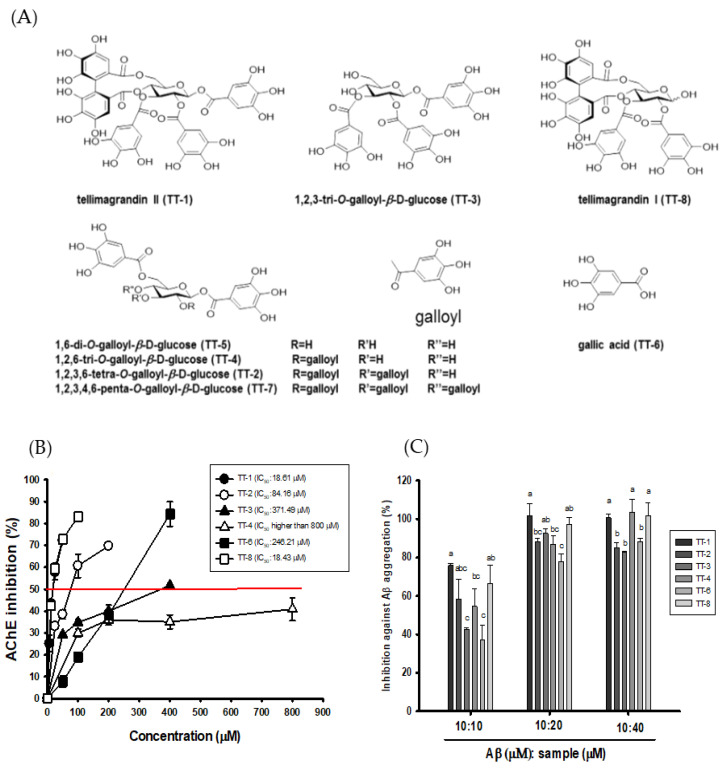
(**A**) The structures of the isolated compounds from TT-hull-50EE; (**B**) Effects of different isolated compounds on acetylcholinesterase (AChE) inhibitory activities; (**C**) Effects of different isolated compounds on anti-Aβ peptide (1–42) aggregations under concentration (μM) ratios of Aβ peptide to the isolated compound of 10:10, 10:20, and 10:40. The quantitative data were presented as mean ± SD of three independent experiments. Multiple group comparisons were performed using one-way analysis of variance (ANOVA) and the *post hoc* Tukey’s test. The different lowercase letters in the anti-Aβ peptide aggregates of purified compounds under the fixed ratio (10:10, 10:20, 10:40) treatment were considered significantly different (*p* < 0.05).

Figure 2A shows the structures of eight isolated compounds from TT-hull-50EE, which involved tellimagrandin II (TT-1) and tellimagrandin I (TT-8) belonging to the ellagitannin class of hydrolysable tannins. The 1,2,3,6-tetra-O-galloyl-β-D-glucopyranoside (TT-2), 1,2,3-tri-O-galloyl-β-D-glucopyranoside (TT-3), 1,2,6-tri-O-galloyl-β-D-glucopyranoside (TT-4), 1,6-di-O-galloyl-β-D-glucopyranoside (TT-5), and 1,2,3,4,6-penta-O-galloyl-β-D-glucopyranoside (TT-7) belonged to the gallotannin class of hydrolysable tannins. The gallic acid (TT-6) was found as a constituent of the other isolated compounds.

Owing to the small amounts of TT-5 and TT-7, only six compounds were used to determine the biological activities. Figure 2B shows the AChE inhibitory activities of these isolated compounds from TT-hull-50EE. Except for TT-4, all isolated compounds showed dose-dependent AChE inhibitory activities, and the IC_50_ of AChE inhibitory activity of TT-1, TT-2, TT-3, TT-6, and TT-8, respectively, was calculated to be 18.6, 84.2, 371.5, 246.2, and 18.4 μM in the present assays. The TT4 at concentration of 800 μM did not reach 50% AChE inhibition; therefore, the IC_50_ of AChE inhibition of TT-4 should be higher than 800 μM. The order of AChE inhibition was TT-8 ≅ TT-1 > TT-2 > TT-6 > TT-3 >> TT-4. The ORAC values of TT-1, TT-2, TT-3, TT-4, TT-6, and TT-8 in vitro at a concentration of 10 μM, respectively, were 5.1, 18.7, 15.1, 18.1, 23.7, and 7.5 μM Trolox equivalents. The ORAC activities of the ellagitannin class of TT-1 and TT-8, respectively, were 50% and 75% of those of Trolox; the ORAC activities of the gallotannin class of TT-2, TT-3, and TT-4 were close to two-fold higher than those of Trolox. The gallic acid (TT-6) showed 2.4-fold higher ORAC activities than those of Trolox. Figure 2C shows the inhibition of Aβ peptide (1-42) aggregation by the isolated compounds at concentration ratios of 1:1, 1:2, and 1:4 of Aβ to the isolated compound. From the results of Figure 2C, it is clear that the isolated compounds showed clear inhibition of Aβ aggregation, and the greater the concentration of the isolated compound that was used, the higher the inhibition of Aβ aggregation that took place. Under the 1:1 ratio of Aβ to isolated compounds at 10 μM, the inhibition of Aβ aggregation of TT-1, TT-2, TT-3, TT-4, TT-6, and TT-8 in vitro was 75.84%, 58.55%, 42.71%, 54.59%, 37.22%, and 66.61%, respectively. Under the 1:2 ratio of Aβ to isolated compounds at 10 μM to 20 μM, the inhibition of Aβ aggregation of TT-1, TT-2, TT-3, TT-4, TT-6, and TT-8 in vitro, respectively, was 100%, 88.08%, 92.44%, 86.82%, 77.73%, and 97.13%. Under the 1:4 ratio of Aβ to isolated compounds at 10 μM to 40 μM, the inhibition of Aβ aggregation of TT-1, TT-2, TT-3, TT-4, TT-6, and TT-8 in vitro, respectively, was 100%, 85.02%, 82.72%, 100%, 88.19%, and 100%. The ellagitannin class of TT-1 and TT-8 showed more potent inhibitory activity against Aβ peptide aggregations and showed significant differences (*p* < 0.05) compared to the other purified compounds.

### 3.3. HPLC Chromatograms of TT-Hull Extracts

**Figure 3:**
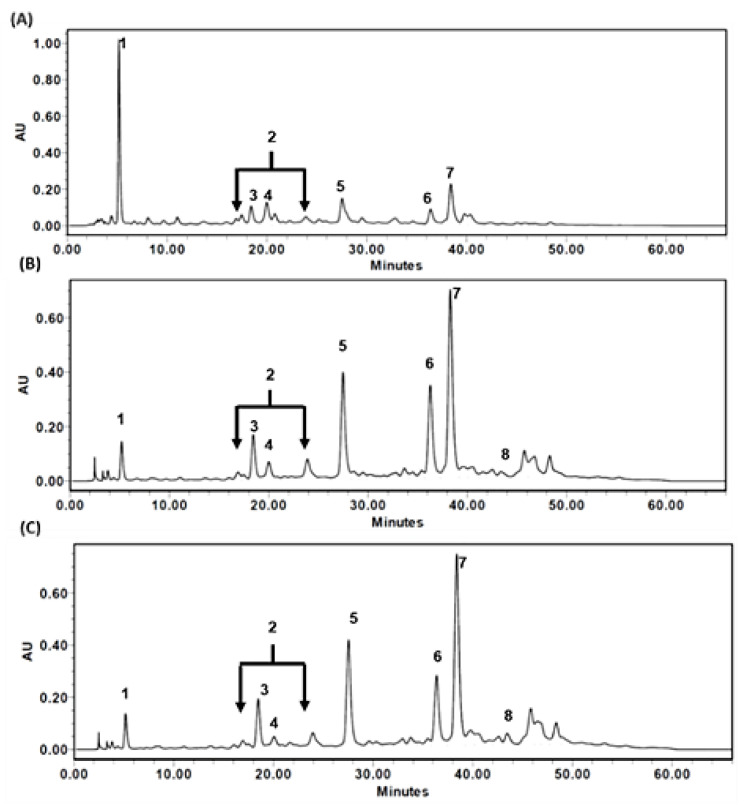
The profiles of HPLC chromatograms of (**A**) TT-hull-HWE; (**B**) TT-hull-50EE; (**C**) TT-hull-95EE. The identified peaks were as follows: Peak 1, gallic acid (as TT-6); Peak 2, tellimagrandin I (as TT-8); Peak 3, 1,6-di-O-galloyl-β-D-glucopyranoside (as TT-5); Peak 4, 1,2,3-tri-O-galloyl-β-D-glucopyranoside (as TT-3); Peak 5, 1,2,6-tri-O-galloyl-β-D-glucopyranoside (as TT-4); Peak 6, tellimagrandin II (as TT-1); Peak 7, 1,2,3,6-tetra-O-galloyl-β-D-glucopyranoside (as TT-2); and Peak 8, 1,2,3,4,6-penta-O-galloyl-β-D-glucopyranoside (as TT-7).

Figure 3 shows the profiles of HPLC chromatograms of TT-hull-HWE (Figure 3A), TT-hull-50EE (Figure 3B), and TT-hull-95EE (Figure 3C) under the same HPLC conditions. The identified peak, based on the elution sequence in each extract, was as follows: Peak 1, gallic acid (as TT-6); Peak 2, tellimagrandin I (as TT-8); Peak 3, 1,6-di-O-galloyl-β-D-glucopyranoside (as TT-5); Peak 4, 1,2,3-tri-O-galloyl-β-D-glucopyranoside (as TT-3); Peak 5, 1,2,6-tri-O-galloyl-β-D-glucopyranoside (as TT-4); Peak 6, tellimagrandin II (as TT-1); Peak 7, 1,2,3,6-tetra-O-galloyl-β-D-glucopyranoside (as TT-2); and Peak 8, 1,2,3,4,6-penta-O-galloyl-β-D-glucopyranoside (as TT-7). All compounds could be found in these three TT-hull extracts, except Peak 8, which was lost in the TT-hull-HWE (Figure 3A).

### 3.4. Effects of the Oral Administration of TT-Hull Extracts on Learning and Memory Functions in Scopolamine-Induced Amnesiac ICR Mice

**Figure 4:**
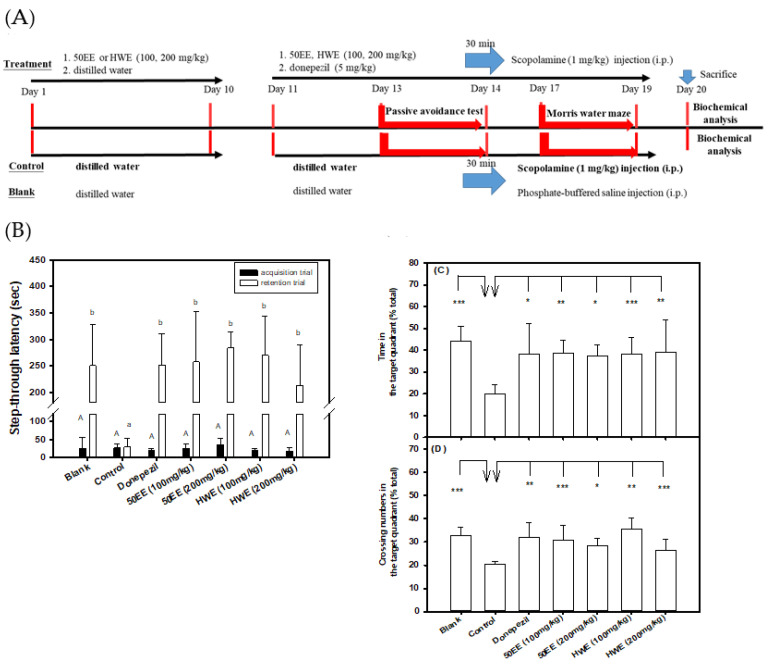
Effects of TT-hull-50EE or TT-hull-HWE (100 and 200 mg/kg) treatments on improvements in learning and memory functions in scopolamine-induced amnesiac mice (six heads/group). (**A**) The experimental protocol, which included four treatment groups, one donepezil-positive control group, one control group, and one blank group; (**B**) the step-through latency (sec) in the passive avoidance test; (**C**) the swimming time in the target quadrant (% of total) in the probe test of the Morris water maze; (**D**) the crossing numbers in the target quadrant (% of total) in the probe test of the Morris water maze. The mice in the control group were injected with scopolamine only in the parallel experiment. Comparisons among multiple groups in the step-through latency (**B**) were performed using one-way analysis of variance (ANOVA) and the *post hoc* Tukey’s test, and the different uppercase letters in the step-through latency in the acquisition trial or the different lowercase letters in the retention trial were considered significantly different (*p* < 0.05) among groups. Student’s t-test was used to compare the treated groups with the control group in the probe test of the Morris water maze (**C**,**D**), and any difference was considered statistically significant when *p* < 0.05 (*), or *p* < 0.01 (**), or *p* < 0.001 (***).

The order of the AChE inhibition of TT-hull extracts was 95EE > HWE > 50EE > C95EE (Figure 1A). The recoveries of C95EE, 95EE, 50EE, and HWE were approximately 8.6%, 6.5%, 10.2%, and 12.8%, respectively. Therefore, TT-hull-50EE and TT-hull-HWE were selected for oral administration in the animal experiments. Figure 4A shows the protocols of the pre-oral administration of TT-hull extracts of 50EE or HWE at concentrations of 100 and 200 mg/kg for ten days, showing preventive functions. From day 11 to day 20, the sample was administered daily; 30 min later, scopolamine was injected for the evaluation of learning and memory functions. The mice in the control group received scopolamine injection only in the parallel experiment. Figure 4B shows the step-through latency (sec) in the passive avoidance test. The step-through latency (sec) in the acquisition trial showed no significant difference (*p* > 0.05) among the groups (the same uppercase letters in the black column). However, the scopolamine-induced amnesiac mice treated with TT-hull-50EE, TT-hull-HWE, and donepezil, and the mice in the blank group, remained for a longer period of time in the light box (the step-through latency) in the retention trial, and all had significant differences (*p* < 0.05) compared to those in the control (lowercase letters in the white column), while the learning and memory functions of mice in the groups treated with TT-hull extracts were improved and comparable to those in the blank group. The different doses of TT-hull-50EE and TT-hull-HWE treatments showed no significant difference (*p* > 0.05) in the step-through latency in the retention trial. Figure 4C,D, respectively, show the time spent in the target quadrant and crossing numbers in the target quadrant in the probe tests of the Morris water maze. Mice in all groups showed no significant differences (*p* > 0.05) in the latency (sec) of the reference memory task of the Morris water maze (data no shown). However, the scopolamine-induced amnesiac mice treated with TT-hull-50EE, TT-hull-HWE, and donepezil, and the mice in the blank group, spent more time in the target quadrant (Figure 4C) and had higher crossing numbers in the target quadrant (Figure 4D), and all showed significant differences (* *p* < 0.05, ** *p* < 0.01, *** *p* < 0.001) compared to those in the control group.

### 3.5. Effects of the Oral Administration of Purified Compounds of TT-1 and TT-2 on Learning and Memory Functions in Scopolamine-Induced Amnesiac ICR Mice

**Figure 5:**
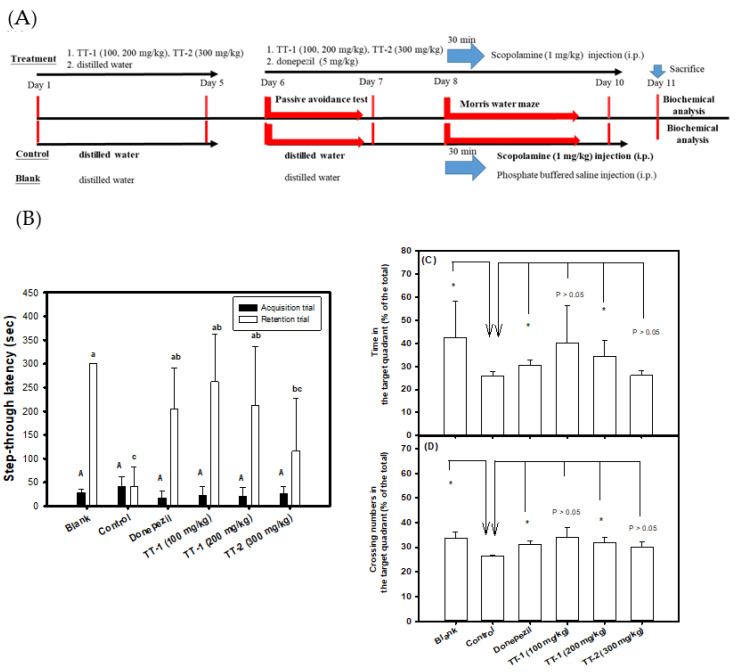
Effects of TT-1 (100 and 200 mg/kg) or TT-2 (300 mg/kg) treatments on improvements in learning and memory functions in scopolamine-induced amnesiac mice (six heads/group). (**A**) The experimental protocol, which included three treatment groups, one donepezil-positive control group, one control group, and one blank group; (**B**) the step-through latency (sec) in the passive avoidance test; (**C**) the swimming time in the target quadrant (% of total) in the probe test of the Morris water maze; (**D**) the crossing numbers in the target quadrant (% of total) in the probe test of the Morris water maze. The mice in the control group were injected with scopolamine only in the parallel experiment. Comparisons among multiple groups in the step-through latency (**B**) were performed using one-way analysis of variance (ANOVA) and the *post hoc* Tukey’s test, and the different uppercase letters in the step-through latency in the acquisition trial or the different lowercase letters in the retention trial were considered significantly different (*p* < 0.05) among groups. Student’s t-test was used to compare treated groups with the control group in the probe test of the Morris water maze (**C**,**D**), and any difference was considered statistically significant when *p* < 0.05 (*).

Figure 5A shows the protocols of the pre-oral administration of purified compounds of TT-1 (100 and 200 mg/kg) and TT-2 (300 mg/kg) for five days. From day 6 to day 10, the sample was administered orally daily; 30 min later, scopolamine was injected; then, evaluation of the learning and memory functions was carried out. Figure 5B shows the step-through latency (sec) in the passive avoidance test. The step-through latency (sec) in the acquisition trial showed no significant difference (*p* > 0.05) among groups (the same uppercase letters in the black column). However, the scopolamine-induced amnesiac mice treated with TT-1 (100 and 200 mg/kg) and donepezil, and the mice in the blank group, but not TT-2 (300 mg/kg), showed longer step-through latency (sec) in the light box of the retention trial and showed significant differences (*p* < 0.05) compared to those in the control (lowercase letters in the white column) group, in which the learning and memory functions of mice in the groups treated with TT-1 were improved and comparable to those in the blank group. The different doses of TT-1 treatment and the donepezil-positive control (5 mg/kg) showed no significant difference (*p* > 0.05) in the step-through latency in the retention trial. Although the TT-2 intervention showed a longer step-through latency (sec) in the retention trial, there were no significant differences (*p* > 0.05) compared to those in the control group. Figure 5C,D, respectively, show the time spent in the target quadrant and crossing numbers in the target quadrant in the probe tests of the Morris water maze. Mice in all groups showed no significant difference (*p* > 0.05) in the latency (sec) of the reference memory task of the Morris water maze (data no shown). However, the scopolamine-induced amnesiac mice treated with a high dose of TT-1 (200 mg/kg) and donepezil, and mice in the blank group, showed a longer time spent in the target quadrant (Figure 5C), higher crossing numbers in the target quadrant (Figure 5D), and significant differences (* *p* < 0.05) compared to those in the control group.

**Figure 6:**
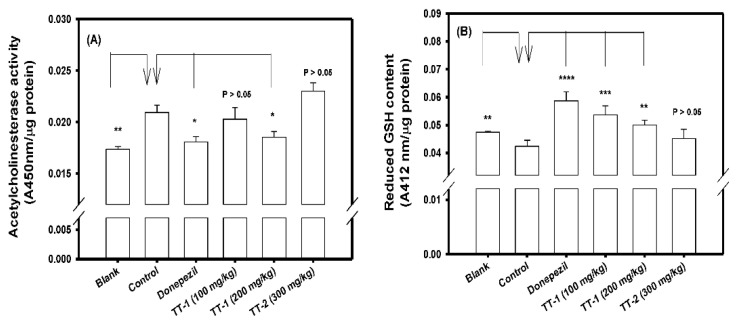
Effects of TT-1 (100 and 200 mg/kg) or TT-2 (300 mg/kg) treatments on (**A**) acetylcholinesterase activity; (**B**) reduced form glutathione (reduced form GSH) contents in brain extracts of the experimental mice. The mice in the control group were injected with scopolamine only (the protocol of the Figure 5A). The quantitative data were presented as mean ± SD of three independent experiments. Student’s t-test was used to compare treated groups with the control group, and any difference was considered statistically significant when *p* < 0.05 (*), or *p* < 0.01 (**), or *p* < 0.001 (***).

Figure 6 shows the AChE activity (Figure 6A) and the reduced form GSH contents (Figure 6B) in the brain extracts of the purified compounds of TT-1, TT-2, and donepezil treatments in the scopolamine-induced amnesiac mice. It was found that the scopolamine-induced amnesiac mice treated with a high dose of TT-1 (200 mg/kg) and donepezil, and the mice in the blank group, showed lower AChE activity (Figure 6A) and higher reduced form GSH contents (Figure 6B), and they showed significant differences (* *p* < 0.05, ** *p* < 0.01, *** *p* < 0.001) compared to those in the control group.

## 4. Discussion

This study is the first in which the oral administration of TT-hull extracts and the isolated compound of TT-1 showed a significant improvement in the learning and memory functions in amnesiac mice models (Figure 4 and Figure 5), compared to those of the control group. The intervention with TT-1 led to lower AChE activities and elevated reduced form GSH contents in the brain extracts of mice subjected to TT-1 treatments, compared to those that received donepezil (Figure 6), which means that the improvement in cognitive symptoms might be correlated with the AChE inhibition and reduced oxidative stress [8,9,10,11]. Therefore, TT-1 might be assumed to be a marker constituent for the quality control of the TT-hull extracts. The extracts of TT-hull-50EE and TT-hull-HWE, and purified compounds TT-1, at the same concentrations of 100 and 200 mg/kg in the present study, showed preventive activities for improving learning and memory function by increasing the step-through latency in the passive avoidance test and time and crossing numbers in the target quadrant in the probe test of the Morris water maze. Based on the profiles of HPLC chromatograms (Figure 3), the area of TT-1 (Peak 6) in the HWE, 50EE, and 95EE accounted in total for approximately 5.02%, 8%, and 7.54%, respectively. It is proposed that the TT-hull extracts showed the synergetic effects of different active ingredients, leading to larger improvements in learning and memory functions compared to the use of one pure compound, such as TT-1, in the amnesiac mice model.

Wang et al. [33] reported that TT-1 was more stable, with the remaining contents being higher than 90% in acidic buffers (pH 2 or pH 4) and the simulated gastric fluids (pH 1.2), compared to that in the neutral and alkaline buffers (pH 8 or pH 10) and the simulated intestinal fluids (pH 7.5). It was estimated that the residual content of TT-1 was reduced to 31.40% in the simulated intestinal fluids at 37 °C for 9 h, and Tuominen and Sundman [34] reported that the ellagitannin class of purified 1,2-digalloyl-4,6-hexahydroxydiphenoyl-β-D-glucopyranose, pedunculagin, and geraniin in the pH 8, 9, 10, and 11 conditions could generate a common compound of ellagic acid via hydrolysis, deprotonation, and oxidative transformation monitored by HPLC/DAD. Gallic acid, with or without the nanoparticle-delivered formulation (10 mg/kg), showed improved learning behavior functions in the probe test of the Morris water maze in scopolamine-induced amnesiac mice, which was attributed to the AChE inhibitory activities [35]. Therefore, TT-1 could generate ellagic acid in vivo, together with gallic acid and other active constituents in the TT-hull extracts, which was shown to ameliorate learning behaviors, and which will need further investigations.

The scopolamine-induced amnesia was used in AD animal models and human clinical trials according to the cholinergic hypothesis, and the scopolamine occupied the muscarinic receptors to block acetylcholine and then temporarily ceased the neurotransmission. The scopolamine-induced amnesia could also enhance the AChE activity and oxidative stress in the brain extracts [30]. The 8-methoxypsoralen (or xanthotoxin) [36], a furanocoumarin isolated from the fruits of Pastinaca sativa, and the demethylcurcumin [30], the minor constituent in naturally occurring curcuminoids, showed the ability to alleviate the learning and memory functions in scopolamine-induced mice by decreasing the AChE activities and improving oxidative stress. Isoflavone of genistein was shown to improve cognitive performance as evaluated by object location recognition and the Morris water maze in scopolamine-induced mice, by decreasing AChE activities and malondialdehyde levels, and increasing SOD activity and GSH content [37]. The learning and memory functions were analyzed either by spatial memory tasks, such as the Morris water maze, or learning tasks in aversive stimuli, such as the passive avoidance task, both of which might be associated with spatial memory in the hippocampus or with communications between the hippocampus and amygdala [38]. It was reported that the brain tissues of AD could display high levels of ROS [39], and Aβ-induced neurotoxicity and/or neurodegenerative disorders were reported to be associated with ROS overproduction [40]. The AGEs were shown to promote ROS production via RAGE [24]. AGEs were also detected in AD amyloid plaques, and the oxidation of glycated proteins might accelerate Aβ accumulation and consequently increase oxidative stress [25]. It was reported that age-related spatial memory impairment could be improved by antioxidants, and the dipeptide Asn-Trp treatments were shown to improve latency (time to reach the platform) in the reference memory task and the probe trial test of the Morris water maze in galactose-induced oxidative stress mice [41]. The Morris water maze was reported to have advantages and disadvantages in its behavior evaluations [42]. It is a form of allocentric (or spatial) navigation characterized by using distal cues compared to egocentric navigation. Therefore, the advantages might include minimal training, reliable learning, and insensitivity to body weight and appetite, and the disadvantages might include stress and the rate of learning in a fixed time. The treated mice showed higher GSH levels and ORAC activities and lower MDA levels in the brain or liver tissues, compared to the control of the galactose-induced group, and the levels of AGEs, including CML and argpyrimidine, in the brain tissues of the treated mice were shown to be significantly lower, compared to the control of the galactose-induced group [41]. The Aβ depositions have been the mainstream theory of AD etiology for the past 30 years, and researchers have developed drugs from small molecules or antibodies based on the processes of Aβ to form Aβ plaques for clinical trials for AD patients [6]. The poor outcomes of AD might be from the multifactorial mechanisms, and no single proposed theory alone could delay AD progression. Therefore, TT-hull extracts (Figure 1) and/or purified compounds (Figure 2) showed AChE inhibitory activities, free radical scavenging activities by ORAC, anti-non-enzymatic protein glycation, and anti-Aβ peptide aggregation in vitro, and they improved the learning and memory functions in scopolamine-induced AD-like amnesiac mice in vivo, which might be beneficial for delaying the onset of AD progression.

In conclusion, the agricultural waste of TT-hulls was recycled to investigate AChE inhibition and then validate the proof-of-concept using scopolamine-induced amnesiac mice, while the learning and memory functions were evaluated by the passive avoidance test and the Morris water maze. The protective functions of TT-hull extracts (such as 50% ethanol extracts and hot water extracts) and the marker constituent TT-1 were shown to improve the learning and memory functions. The Japanese water caltrop hull extracts were shown to lower the blood glucose in fasting mice by an oral carbohydrate tolerance test [29]. Therefore, the TT-hull has the potential to be developed as a functional food or to satisfy unmet medical needs, for the treatment of degenerative disorders.

## Data Availability

All figures and data used to support this study are included within this article.
